# Cumulative Life Course Impairment in Atopic Dermatitis: A Comprehensive Review

**DOI:** 10.3390/medsci14040499

**Published:** 2026-08-20

**Authors:** Claudio Brescia, Chiara Palagiano, Martina Turco, Luca Potestio, Francesca di Vico, Maddalena Napolitano

**Affiliations:** Section of Dermatology, Department of Clinical Medicine and Surgery, University of Naples Federico II, Via Pansini 5, 80131 Naples, Italy

**Keywords:** atopic dermatitis, cumulative life course impairment, quality of life

## Abstract

**Background**: Atopic dermatitis (AD) is a chronic inflammatory skin disease with a relapsing course and a substantial multidimensional burden. Conventional clinical and patient-reported outcome measures mainly provide cross-sectional evaluations and may not fully capture the long-term impact of the disease. The concept of cumulative life course impairment (CLCI) has been proposed to describe the progressive and lifelong consequences of chronic dermatologic conditions. **Methods**: This narrative review is based on a non-systematic literature search conducted in PubMed/MEDLINE and Embase up to July 2026. Relevant studies addressing quality of life, mental health, comorbidities, and economic burden in AD were identified and narratively synthesized, with particular focus on evidence supporting the CLCI framework. **Results**: AD exerts a cumulative burden across multiple domains. Pruritus and sleep disturbance emerge as key drivers, contributing to a self-reinforcing cycle involving psychological distress and impaired daily functioning. Increased rates of anxiety and depression, along with social stigmatization, negatively affect interpersonal relationships, educational attainment, and work productivity. Comorbidities and the chronic relapsing nature of AD further amplify long-term impairment. Economic burden, including both direct and indirect costs, acts as both a consequence and a driver of cumulative disadvantage. Recurrent disease flares play a central role in sustaining and amplifying this trajectory over time. **Conclusions**: AD should be considered a life-course disease in which interacting biological, psychological, and social factors shape long-term outcomes. A CLCI-oriented approach may improve patient stratification and support earlier, multidisciplinary, and proactive management strategies aimed at reducing long-term burden and improving overall outcomes.

## 1. Introduction

Atopic dermatitis (AD) is the most common chronic inflammatory skin disease worldwide and represents a major burden for patients and healthcare systems [1]. Although approximately 85% of cases develop before the age of five and many patients experience partial or complete remission during adolescence, AD may persist into adulthood or arise de novo later in life [2,3,4]. In this regard, two major clinical patterns have been described: forms that begin in childhood and persist into adult life, commonly referred to as paediatric-onset atopic dermatitis (POAD), and forms that first appear in adulthood, known as adult-onset atopic dermatitis (AOAD) [5]. Prevalence estimates of adult AD vary across epidemiological studies, reflecting differences in diagnostic criteria and study populations, but the condition is increasingly recognized as a significant health problem beyond childhood [3,4].

The clinical assessment of AD traditionally relies on clinician-reported outcomes (ClinROs), including the Eczema Area and Severity Index (EASI), Scoring Atopic Dermatitis (SCORAD), and Investigator’s Global Assessment (IGA), alongside patient-reported outcomes (PROs) such as the Patient-Oriented Eczema Measure (POEM), Dermatology Life Quality Index (DLQI), and pruritus intensity scales (e.g., Numerical Rating Scale, NRS) [6]. While these instruments are essential for evaluating disease activity and guiding therapeutic decisions, they primarily provide a cross-sectional assessment of disease burden.

Beyond clinical signs, AD exerts a profound and multidimensional impact on patients’ quality of life (QoL), often only partially captured by traditional outcome measures. Persistent pruritus, sleep disturbance, visible skin lesions, and the need for continuous treatment substantially impair daily functioning, emotional well-being, and social interactions [7,8,9,10]. Patients frequently report anxiety, depression, stigmatization, and reduced self-esteem, highlighting the significant psychosocial burden associated with the disease [8,11]. Among symptoms, sleep disruption—largely driven by nocturnal itch—represents one of the most disabling features, contributing to fatigue, reduced productivity, and impaired cognitive performance [9,12]. In addition, AD negatively affects educational attainment, work performance, and interpersonal relationships, generating considerable indirect socioeconomic costs and impacting caregivers and family members, particularly in pediatric cases [10,13,14]. Importantly, QoL impairment may persist even during periods of apparent clinical control, suggesting that the burden of AD extends beyond measurable disease activity and accumulates over time [15]. This discrepancy between clinical severity and lived disease experience underscores the need to move beyond cross-sectional assessments and consider the long-term, cumulative consequences of the disease.

Cumulative life course impairment (CLCI) refers to the progressive accumulation of disease-related disadvantages and potentially irreversible life changes over the course of a chronic condition. Unlike conventional quality-of-life instruments, which primarily capture current or recent disease burden, the CLCI framework considers how disease activity, duration, age at onset, recurrent flares, treatment burden, comorbidities, psychological vulnerability, stigma, coping resources, and socioeconomic context interact over time to alter health, educational, occupational, relational, and financial trajectories [16]. Therefore, comparable levels of current clinical severity may result in markedly different long-term consequences depending on the timing and duration of disease exposure and on the presence of protective factors such as effective treatment, social support, and adaptive coping. Two dermatology-specific instruments have been developed to operationalize this framework in adults [16,17,18,19]. The DermCLCI-r retrospectively assesses impairments and life-changing consequences experienced throughout the disease course, whereas the DermCLCI-p evaluates current burden and domains associated with future risk. Both instruments contain 30 items covering symptoms, psychological and physical impairment, comorbidities, occupational functioning, treatment burden, sexuality, family and social relationships, everyday functioning, and financial consequences [16,17,18,19]. However, their longitudinal and disease-specific validation in AD remains limited, and no dedicated validated instrument is currently available for children and adolescents. The application of CLCI to AD has been explored in only a limited number of publications. An early conference report evaluated patient experiences to determine whether the impact of AD appeared cumulative, whereas the recent review by Augustin et al. provided a broad lifespan-oriented overview focused on the recognition, measurement, and prevention of CLCI and highlighted the need for a dedicated paediatric assessment instrument [17,18]. However, an important knowledge gap remains: it is still unclear to what extent the available empirical evidence demonstrates cumulative, temporally evolving impairment rather than multidimensional disease burden measured at a single time point.

The present review addresses this gap through an evidence-focused critical appraisal of primary studies, meta-analyses, and real-world evidence across symptom-related, psychiatric, cognitive, social, educational, occupational, comorbidity, caregiver, economic, flare-related, and treatment-related domains. Rather than proposing an alternative conceptual framework, we examine whether the available evidence supports persistence, recurrence, interaction, temporal accumulation, or lasting life-course consequences. Particular attention is given to age at onset, life-stage-specific vulnerability, recurrent flares as a possible mechanism of accumulation, and the dual role of treatment as both a potential modifier and a contributor to CLCI. This approach aims to distinguish established associations from plausible but still unproven cumulative pathways and to identify the specific longitudinal evidence required to validate CLCI in AD.

## 2. Materials and Methods

This narrative review was conducted through a non-systematic literature search performed in PubMed/MEDLINE and Embase databases up to July 2026. The search strategy included combinations of the following keywords: “atopic dermatitis” AND “comorbidities”, “economic burden”, “quality of life”, “mental health”, and “cumulative life course impairment”. Priority was given to systematic reviews and meta-analyses, longitudinal cohort studies, large population-based or cross-sectional studies, international guidelines, and studies specifically addressing CLCI or long-term disease trajectories. Because evidence directly measuring CLCI in AD remains limited, studies evaluating individual domains potentially contributing to cumulative impairment were also included and interpreted within the CLCI framework. Additional relevant articles were identified through manual screening of the reference lists of selected papers, including review articles and international clinical guidelines. Studies were selected based on their relevance to the aims of the review, with particular emphasis on clinical practice guidelines, randomized controlled trials, real-world evidence studies, and high-quality systematic reviews.

The retrieved literature was critically appraised and narratively synthesized to reflect current evidence on the multidimensional burden of atopic dermatitis, including clinical severity, psychosocial impact, and long-term consequences. Particular attention was given to studies employing validated clinical and patient-reported outcome measures (e.g., EASI, SCORAD, IGA, POEM, DLQI, pruritus-NRS), as well as to investigations addressing quality of life impairment, mental health comorbidities, and socioeconomic burden.

Seminal publications on cumulative life course impairment (CLCI), along with key studies on chronic inflammatory dermatoses and atopic dermatitis, were specifically included to contextualize the long-term impact of the disease. References were prioritized according to scientific relevance, methodological quality, and their contribution to understanding the cumulative and multidimensional burden of AD. The evidence was synthesized according to the principal domains of the CLCI framework, including disease-related symptoms, psychological and cognitive outcomes, social and relational functioning, educational and occupational consequences, comorbidities, treatment and economic burden, and the influence of age at onset and life stage. Particular attention was paid to distinguishing cross-sectional burden from evidence of persistence, accumulation, or life-changing consequences over time. Given the narrative nature of the review, no formal risk-of-bias assessment or meta-analytic synthesis was performed.

## 3. Results

### 3.1. The CLCI Framework and Its Assessment in Atopic Dermatitis

As outlined in the Introduction, the central question is not whether atopic dermatitis is associated with a substantial disease burden, but whether this burden persists, recurs, interacts across different domains, or produces lasting changes over time. AD-specific evidence directly evaluating these cumulative processes remains limited. An early conference report explored patient experiences to determine whether AD-related impairment appeared cumulative, although the limited information available from the abstract did not permit a detailed assessment of its methods and findings [18]. More recently, Augustin et al. proposed a lifespan-oriented interpretation of CLCI in AD and emphasized the importance of identifying patients at risk, incorporating CLCI assessment into clinical practice, and developing age-appropriate assessment instruments [17]. The first study directly applying the CLCI-R and CLCI-P instruments in AD included 82 adults with moderate-to-severe disease. Retrospective impairment was observed in patients with both moderate and severe AD, whereas prospective impairment was significantly higher among patients with severe disease. CLCI-P scores correlated with DLQI and clinical severity, and greater prospective impairment was associated with higher DLQI, previous cumulative impairment, and a disease duration of at least 21 years [20]. These findings provide initial support for the applicability of CLCI assessment in AD. However, the cross-sectional and single-centre design of the study does not establish temporal progression, causality, or reversibility. Accordingly, in the sections below, cross-sectional associations are interpreted as evidence of multidimensional disease burden. Evidence is considered supportive of CLCI only when it demonstrates persistence, recurrence, temporal accumulation, interaction across burden domains, occurrence during sensitive life stages, or lasting consequences for health, education, employment, relationships, or financial stability.

### 3.2. Age at Onset and CLCI Across Different Life Stages

The timing of AD onset may substantially influence the nature and magnitude of CLCI. Pediatric-onset disease is characterized by prolonged exposure to symptoms and treatment demands during critical stages of physical, emotional, cognitive, and social development. By contrast, adult-onset AD may disrupt already-established occupational, relational, and health trajectories. Biological differences have also been identified between persistent pediatric-onset and adult-onset AD, supporting the existence of partly distinct disease profiles rather than a uniform condition differing only in duration [5].

During infancy and childhood, pruritus, sleep disruption, recurrent infections, treatment demands, and atopic comorbidities may affect growth, behaviour, school attendance, and participation in age-appropriate activities. Because young children depend on their families for treatment and symptom management, the burden frequently extends to parents and caregivers, who may experience sleep loss, emotional distress, reduced work productivity, and increased financial demands [14,21]. The development of asthma, allergic rhinitis, or food allergy may further increase healthcare use and management complexity during childhood [22,23,24].

Adolescence represents a particularly sensitive period because visible skin disease and treatment requirements may interfere with body image, autonomy, identity formation, peer relationships, and treatment adherence. Stigma, embarrassment, bullying, and avoidance behaviours may reduce social participation and psychological resilience, while sleep disturbance and disease-related distress may negatively affect educational performance [25,26]. These effects may become cumulative when they influence educational choices, self-confidence, or the development of long-term social relationships.

In adulthood, persistent or recurrent AD may affect employment, career progression, intimate relationships, family planning, and financial stability [27,28,29,30,31]. Evidence from the Scars of Life project suggests that adults whose AD began in childhood may report greater long-term impairment than those with adult-onset disease, supporting the hypothesis that prolonged exposure during sensitive developmental periods has lasting consequences [32]. Nevertheless, adult-onset AD should not be regarded as inherently less burdensome, as its onset may coincide with major occupational and family responsibilities and may be associated with distinct systemic and inflammatory characteristics [5].

In older adults, CLCI may be influenced by multimorbidity, polypharmacy, reduced physical resilience, social isolation, sleep disturbance, and cognitive decline. The coexistence of AD with cardiovascular, metabolic, psychiatric, autoimmune, or other age-related conditions may complicate therapeutic decisions and increase treatment burden [4]. However, direct longitudinal comparisons of CLCI between pediatric-onset and adult-onset AD and across different life stages remain limited. Current evidence therefore supports the existence of potentially different CLCI trajectories but does not yet permit their precise quantification.

Overall, current evidence suggests that age at onset and life stage may shape different CLCI trajectories. However, direct longitudinal comparisons between paediatric-onset and adult-onset AD and across different life stages remain insufficient.

### 3.3. Pruritus and Sleep Disturbance

Pruritus is the hallmark symptom of AD and one of the most important determinants of disease burden [33,34]. Its pathophysiology involves complex interactions among keratinocytes, immune cells, the epidermal barrier, and peripheral sensory nerves. Cytokines such as interleukin-31, together with proteases, histamine-independent pathways, neuropeptides, and neuronal sensitization, contribute to the itch–scratch cycle and perpetuate cutaneous inflammation [34,35,36].

Persistent pruritus has consequences extending beyond physical discomfort. It interferes with concentration, daily activities, emotional well-being, social functioning, and work productivity and is consistently associated with poorer health-related quality of life [37]. Its cumulative relevance is particularly evident when symptoms begin early, recur frequently, or remain inadequately controlled for prolonged periods, thereby repeatedly disrupting developmental, educational, relational, or occupational activities.

Sleep disturbance is closely linked to pruritus and represents another major contributor to long-term burden. Patients with AD may experience prolonged sleep latency, recurrent nocturnal awakenings, reduced sleep duration, and poor sleep quality, largely because of nocturnal itch, scratching, and circadian changes in inflammatory activity and skin barrier function [38,39]. The association is bidirectional: pruritus disrupts sleep, whereas sleep deprivation may increase symptom perception, impair emotional regulation, and promote inflammatory responses [40,41].

Repeated sleep disruption may result in daytime fatigue, impaired attention and cognitive performance, reduced school or workplace productivity, and diminished psychological resilience. Evidence from the broader sleep literature also links chronic sleep deprivation with systemic inflammation, metabolic dysregulation, cardiovascular risk, anxiety, and depression [42,43,44,45,46,47]. Although these associations are not specific to AD and do not prove that AD-related sleep disturbance directly causes these outcomes, they support the biological and clinical plausibility of sleep acting as an important mediator of cumulative impairment. The itch–sleep axis may therefore contribute to CLCI when repeated nocturnal symptoms produce persistent consequences that are not completely reversed during periods of improved skin disease. Thus, pruritus and sleep disturbance are well-established components of the multidimensional burden of AD. Their contribution to CLCI remains mainly supported by biological plausibility and indirect evidence, as longitudinal confirmation of persistent or irreversible consequences is still limited.

### 3.4. Mental Health and Psychological Vulnerability

Mental health impairment represents a central domain of CLCI in AD. Systematic reviews and meta-analyses have consistently demonstrated increased rates of depression, anxiety, psychological distress, and suicidal ideation in both adult and pediatric populations [48,49]. In adults, AD has been associated with approximately twofold higher odds of both depression and anxiety compared with individuals without AD, with pooled odds ratios of 2.19 for each condition [48].

The relationship between AD and psychiatric morbidity is likely multifactorial and bidirectional. Persistent pruritus, sleep deprivation, visible lesions, stigma, treatment frustration, and restrictions in daily activities may contribute to emotional distress. Conversely, psychological stress, depression, and anxiety may increase symptom perception, reduce treatment adherence, and exacerbate disease activity through neuroimmune and neuroendocrine mechanisms. Sleep disturbance represents an important mediator within this relationship, but psychiatric impairment may also occur independently of measured clinical severity.

Studies using validated psychometric instruments have documented greater depressive symptoms, anxiety, reduced self-esteem, social withdrawal, and impaired psychological well-being among patients with AD [49,50]. Psychiatric burden may be further amplified by atopic and non-atopic multimorbidity, which increases treatment complexity and the perception of poor overall health [51]. During childhood and adolescence, psychological vulnerability may interfere with social development and educational attainment, while in adults it may affect interpersonal relationships, occupational functioning, and resilience [25,26].

Nevertheless, much of the available evidence is cross-sectional. It therefore remains uncertain whether psychiatric disorders arise as consequences of AD, contribute to worsening disease, share underlying biological or socioeconomic determinants, or reflect a combination of these mechanisms. Within the CLCI framework, mental health conditions are most relevant when they persist beyond individual flares, interact with other burden domains, or produce durable changes in social, educational, relational, or occupational trajectories. Accordingly, the association between AD and mental health impairment is well established. However, direct evidence that psychological impairment accumulates over time and produces lasting changes in educational, relational, or occupational trajectories remains limited.

### 3.5. Cognitive Outcomes

Cognitive impairment may represent an additional but insufficiently characterized domain of CLCI. In children, adolescents, and working-age adults, nocturnal pruritus, fragmented sleep, fatigue, anxiety, and depression may impair concentration, memory, executive functioning, and academic or occupational performance [25,38,39,40,41]. These potentially reversible functional effects should, however, be distinguished from persistent cognitive decline and neurodegenerative outcomes in middle-aged and older adults.

A systematic review and meta-analysis including five cohort studies, 15 study arms, and more than 8.5 million participants evaluated the relationship between AD and cognitive dysfunction in middle-aged and older individuals. AD was associated with a modestly increased risk of all-cause dementia, with a pooled hazard ratio of 1.16, and Alzheimer-type dementia, with a pooled hazard ratio of 1.28. The pooled estimate for vascular dementia was not statistically conclusive because the confidence interval included unity [52].

These results should be interpreted cautiously. The included studies differed in geographic setting, AD ascertainment, cognitive outcome definitions, study design, and adjustment for potential confounders. Considerable heterogeneity was reported for some outcomes, and observational data cannot establish causality. Sleep disturbance, psychiatric disease, cardiovascular risk, socioeconomic factors, medication exposure, systemic inflammation, and healthcare-use patterns may partly mediate or confound the observed associations [52]. Cognitive outcomes are therefore relevant to a life-course perspective, but further longitudinal studies are required to determine whether AD independently contributes to cognitive decline and whether effective long-term disease control modifies this risk. Current data therefore support a possible long-term cognitive burden associated with AD but do not yet establish a causal or cumulative CLCI pathway.

### 3.6. Social, Relational, Educational, and Occupational Impairment

Social, relational, educational, and occupational consequences represent the functional translation of persistent AD symptoms into altered life opportunities. Large cross-sectional and real-world studies have demonstrated reduced work productivity, increased absenteeism and presenteeism, and greater overall activity impairment, particularly among patients with moderate-to-severe disease [27,28]. Pruritus, sleep disturbance, visible lesions, treatment demands, and psychological distress may each contribute to reduced concentration, impaired performance, and absence from school or work.

The cumulative impact extends beyond individual episodes of reduced productivity. Repeated school absence, difficulty concentrating, or social avoidance during childhood and adolescence may influence educational attainment and subsequent career opportunities. In adults, recurrent flares and treatment-related demands may limit career progression, occupational flexibility, and financial security. However, longitudinal studies directly linking AD with completed education, career advancement, or lifetime earnings remain comparatively scarce.

Visible lesions and perceived stigma may lead to embarrassment, avoidance behaviours, social withdrawal, and reduced self-esteem [53]. The long-term effects of stigmatization may be particularly relevant when AD begins during childhood or adolescence, as repeated negative social experiences during these periods may influence identity formation and future interpersonal behaviour [16,53]. Psychological distress may mediate part of this relationship, although social impairment is also independently influenced by disease visibility, cultural attitudes, socioeconomic resources, and the availability of social support.

Intimate and sexual relationships represent an additional and frequently underrecognized domain. Patients with AD may report impaired body image, reduced sexual desire, avoidance of physical contact, and sexual dysfunction, particularly in the presence of extensive or severe disease [29,30,31]. These consequences may affect relationship satisfaction and the formation or maintenance of intimate partnerships. Within the CLCI framework, social and relational impairment becomes cumulative when recurrent disease experiences result in persistent withdrawal, missed developmental opportunities, or lasting changes in educational, occupational, or interpersonal trajectories. These findings strongly support the presence of multidimensional social and functional burden. However, evidence directly demonstrating persistent changes in educational attainment, employment progression, lifetime income, or relationships remains comparatively limited.

### 3.7. Economic and Treatment Burden

AD is associated with substantial direct and indirect costs [8,54,55]. Direct costs include medical consultations, pharmacological treatments, hospitalizations, diagnostic procedures, and transportation. Indirect costs arise from absenteeism, presenteeism, reduced school or work productivity, caregiver responsibilities, and loss of occupational opportunities. Both components generally increase with disease severity and chronicity [8,54].

Out-of-pocket expenditure may include emollients, hygiene products, non-prescription medications, clothing, cleaning products, and complementary treatments [56,57,58,59]. These costs may be especially relevant in healthcare systems with incomplete reimbursement and may contribute to unequal access to effective care. In pediatric AD, economic burden extends to the family, as caregivers may reduce working hours, take leave, or experience impaired productivity because of medical appointments, nocturnal caregiving, and daily treatment requirements [14,21].

Treatment burden is not limited to financial expenditure. Time-consuming skincare routines, repeated topical applications, monitoring requirements, concerns about adverse effects, and the need to escalate or change treatment may interfere with everyday activities and contribute to treatment fatigue. Difficulties accessing specialist care and effective therapies may prolong uncontrolled disease and indirectly increase burden across other CLCI domains [17].

Current evidence therefore supports a substantial economic and treatment burden associated with AD. However, longitudinal data remain insufficient to determine whether these costs progressively alter employment, financial security, access to care, or other life-course trajectories.

### 3.8. Atopic and Non-Atopic Comorbidities

AD is associated with a broad spectrum of atopic and non-atopic comorbidities and is increasingly recognized as a systemic inflammatory disease [60]. Within a CLCI framework, comorbidities can function as burden multipliers by increasing symptom load, treatment complexity, healthcare use, and restrictions on daily activities.

Asthma and allergic rhinitis are among the most frequent atopic comorbidities. A systematic review and meta-analysis estimated a pooled asthma prevalence of 25.7% among patients with AD compared with 8.1% among reference individuals, corresponding to an odds ratio of 3.03 [22]. Similarly, pooled rhinitis prevalence was 40.5% in patients with AD and 18.0% in individuals without AD, with an overall odds ratio of 3.00 [23].

Food sensitization and food allergy are particularly relevant in childhood and in patients with more severe AD. Skin barrier dysfunction may facilitate epicutaneous allergen exposure and sensitization, while food-related concerns and elimination diets may further affect nutrition, family routines, anxiety, and quality of life [24]. A large systematic review and meta-analysis reported pooled prevalences of food sensitization and food allergy of 48.4% and 32.7%, respectively, among individuals with AD, although estimates varied according to age, disease severity, diagnostic method, and study population [61]. These values must be interpreted carefully because sensitization, self-reported food allergy, and challenge-confirmed food allergy are not interchangeable clinical entities.

The relationship between AD and allergic contact disease is more nuanced. Barrier impairment and repeated exposure to topical preparations may increase the clinical relevance of contact allergy in selected patients. However, an updated systematic review and meta-analysis found no significant overall association between AD and contact sensitization, with a pooled odds ratio of 1.08. A possible association was observed in children and adolescents and for selected allergens, whereas no clear overall association was identified in adults [62]. These findings support targeted patch testing when suggested by the clinical history, treatment resistance, or lesion distribution rather than considering contact sensitization an inevitable component of AD.

Emerging evidence also suggests associations between AD and cardiovascular, metabolic, autoimmune, infectious, and psychiatric conditions [63]. However, the strength and causality of these relationships vary, and residual confounding related to disease severity, treatment, lifestyle, socioeconomic status, and healthcare utilization cannot be excluded. Comorbidities contribute most clearly to CLCI when they develop sequentially or coexist over prolonged periods, increase management complexity, or interact with sleep disturbance, mental health, occupational functioning, and economic burden. Comorbidities may therefore act as multipliers of multidimensional burden. Their contribution to CLCI is most plausible when they persist, develop sequentially, interact with other burden domains, and produce long-term consequences.

### 3.9. Disease Flares and Long-Term Control

AD is characterized by a chronic relapsing course, and recurrent flares may represent a major mechanism through which impairment accumulates over time. Although a universally accepted definition remains lacking, flares are generally understood as acute worsening of signs and symptoms requiring treatment initiation or escalation. Potential triggers include microbial dysbiosis and *Staphylococcus aureus* colonization, environmental allergens, irritants, climatic conditions, sweating, psychological stress, and insufficient maintenance treatment [64,65]. At the biological level, flares reflect dynamic interactions between epidermal barrier dysfunction, type 2 inflammation, and additional immune pathways [66,67,68].

Each flare may cause a temporary but clinically meaningful increase in pruritus, sleep disturbance, pain, psychological distress, and treatment burden. Recurrent episodes may interrupt school, work, social activities, and interpersonal relationships. The unpredictability of flares may additionally produce anticipatory anxiety, avoidance behaviours, and difficulties planning occupational or leisure activities.

From a CLCI perspective, the importance of flares lies not only in their frequency or severity but also in the possibility of repeated disruption followed by incomplete recovery. A single flare may produce transient impairment, whereas recurrent or prolonged flares during important developmental, educational, relational, or occupational transitions may generate lasting consequences. Their cumulative effects may also interact with psychiatric vulnerability, comorbidities, financial burden, and reduced resilience.

Biologics and Janus kinase inhibitors have improved control of inflammation, pruritus, and disease exacerbations in moderate-to-severe AD [58,59]. Achieving sustained disease stability and reducing flare frequency may therefore represent a plausible strategy for interrupting the accumulation of impairment. However, direct evidence that flare prevention modifies long-term CLCI remains limited. Longitudinal studies combining clinical outcomes with CLCI-specific measures are required to determine whether early and sustained disease control can prevent, reduce, or reverse cumulative impairment. Recurrent flares therefore represent a plausible mechanism through which impairment may accumulate. However, direct evidence demonstrating that flare prevention reduces, prevents, or reverses CLCI remains lacking.

## 4. Discussion

The present review supports the relevance of applying a cumulative life course impairment framework to AD. However, it also highlights an important distinction between multidimensional disease burden and demonstrated cumulative impairment. Most available studies document associations between AD and individual clinical, psychological, cognitive, social, or economic outcomes at a single point in time. By contrast, CLCI implies that these effects persist, recur, interact, or occur during sensitive life stages, ultimately producing lasting or potentially irreversible changes in an individual’s health, relationships, education, employment, or financial trajectory [69,70,71,72]. Therefore, not every impairment associated with AD should automatically be considered cumulative; its temporal course, interaction with other vulnerabilities, and long-term consequences must also be considered.

The CLCI framework may complement conventional ClinROs and PROs by incorporating dimensions that are incompletely captured by instruments focused on current disease activity or recent quality of life. The development of the DermCLCI-r and DermCLCI-p represents an important step towards the operational assessment of retrospective impairment and future CLCI risk [19]. Initial application of these instruments in adults with moderate-to-severe AD has provided preliminary evidence of their clinical relevance, particularly in patients with severe, longstanding disease and greater current quality-of-life impairment. Nevertheless, validation specifically in AD remains limited, and evidence regarding responsiveness to treatment, clinically meaningful thresholds, predictive accuracy, and longitudinal change is still required. Moreover, no dedicated validated CLCI instrument is currently available for children and adolescents, despite the particular vulnerability of these populations to developmental, educational, and familial consequences.

Taken together, the findings reviewed suggest that CLCI in AD is unlikely to result from any single symptom or comorbidity. Rather, age at onset and life stage may modify the effects of recurrent pruritus, sleep disruption, psychological distress, cognitive and functional impairment, stigma, and treatment demands. The cumulative relevance of these factors depends not only on their presence, but also on their persistence, recurrence, interaction, timing during sensitive developmental or life transitions, and the extent to which recovery remains incomplete between disease flares. This interpretation further supports the need to distinguish contemporaneous multidimensional burden from impairment that progressively alters subsequent health, educational, occupational, or relational opportunities. These findings also raise questions regarding the content validity of the currently available dermatology-specific CLCI instruments in AD. The DermCLCI-r and DermCLCI-p include broad domains covering symptoms, psychological and physical impairment, comorbidities, occupational functioning, treatment burden, sexuality, family and social relationships, everyday functioning, and financial consequences [19]. However, because these instruments were developed for chronic dermatological diseases in general, it remains uncertain whether they adequately capture AD-specific pathways, including nocturnal pruritus and sleep disruption, recurrent and unpredictable flares, anticipatory anxiety, cognitive and educational consequences, and the practical burden of prolonged topical or systemic treatment. The inclusion of broad symptom or family-related domains does not necessarily ensure sufficient assessment of these specific experiences. This limitation may be particularly relevant in paediatric AD, for which no dedicated validated CLCI instrument is currently available. A future age-appropriate instrument may benefit from linked but distinct child- and caregiver-reported components. The child component could assess sleep, development, school participation, peer relationships, body image, autonomy, and missed age-specific opportunities, whereas the caregiver component could assess sleep deprivation, emotional distress, treatment workload, work productivity, healthcare demands, and financial consequences. Maintaining separate but complementary components would distinguish the child’s own cumulative impairment from the spillover burden experienced by the family while capturing their potentially interdependent trajectories. Importantly, such an instrument should not simply reproduce a paediatric quality-of-life questionnaire, but should assess whether impairments persist, recur, occur during sensitive life stages, result in missed opportunities, or generate lasting consequences. Dedicated qualitative and longitudinal content-validation studies involving children, adolescents, and caregivers are therefore required.

Social, relational, educational, and occupational outcomes represent the points at which repeated symptoms may translate into lasting life disadvantage. Cross-sectional and real-world studies consistently show increased absenteeism, presenteeism, activity impairment, social withdrawal, perceived stigma, and interference with intimate relationships [26,27,28,29,30,31]. However, evidence directly linking AD with completed educational attainment, long-term career progression, lifetime income, or persistent relational outcomes remains comparatively limited. Future studies should distinguish temporary functional impairment during active disease from changes that persist after clinical improvement or alter subsequent opportunities.

The CLCI framework may also extend beyond the individual patient. In paediatric AD, caregivers frequently experience sleep deprivation, psychological distress, reduced work productivity, treatment-related demands, and financial burden [73]. These consequences may influence the child’s disease trajectory through caregiver stress, treatment adherence, access to care, and household resources, supporting the potential interdependent or intergenerational dimension discussed above.

Atopic and non-atopic comorbidities may further accelerate cumulative impairment. Asthma, allergic rhinitis, and food allergy can increase symptom burden, healthcare utilization, treatment complexity, and restrictions on everyday. However, these conditions should not be interpreted as uniform or inevitable components of AD. In particular, food sensitization must be distinguished from clinically confirmed food allergy, and the relationship between AD and contact sensitization varies according to age, clinical setting, and individual allergens. Associations with cardiovascular, metabolic, autoimmune, infectious, and psychiatric conditions also require cautious interpretation because disease severity, systemic inflammation, lifestyle, socioeconomic status, treatment exposure, and surveillance bias may contribute.

Treatment may play a dual role within the CLCI framework. Effective and sustained disease control has the potential to reduce pruritus, sleep disruption, flares, psychosocial distress, healthcare use, and interference with daily activities. Consequently, timely access to appropriate therapy may prevent the accumulation of avoidable impairment. However, treatment itself may also contribute to cumulative burden through adverse effects, monitoring requirements, treatment fatigue, fear of toxicity, financial costs, and difficulties accessing specialist care. Treatment-related CLCI should therefore be considered alongside disease-related impairment rather than assuming that all treatment exposure is protective [17,19].

For topical therapies, prolonged or inappropriate use of potent topical corticosteroids may be associated with skin atrophy, striae, telangiectasia, and local adverse effects, particularly when used continuously on sensitive anatomical sites. Nevertheless, the available long-term evidence is limited and does not support the assumption that correctly prescribed, intermittent topical corticosteroid use inevitably produces clinically relevant skin damage [74]. Excessive concern about topical corticosteroid toxicity may itself lead to undertreatment, poor adherence, persistent disease activity, and potentially greater cumulative impairment [74]. The risk–benefit assessment should therefore distinguish appropriate proactive or intermittent use from prolonged, unsupervised, or high-potency exposure [74]. Systematic reviews confirm that evidence on long-term topical corticosteroid safety is limited, while intermittent regimens appear substantially less hazardous than commonly perceived.

Systemic corticosteroids are particularly relevant because repeated or prolonged exposure may be associated with rebound flares, gastrointestinal complications, metabolic effects, hypertension, infections, adrenal suppression, osteoporosis, osteonecrosis, ocular complications, and psychological adverse effects [75,76,77]. For these reasons, current guidelines recommend against their routine or long-term use in AD, except for carefully selected short-term circumstances or as a bridge to more appropriate treatment [78,79,80,81]. Conventional systemic immunosuppressants also carry treatment-specific burdens: ciclosporin requires monitoring for nephrotoxicity and hypertension, while methotrexate, azathioprine, and mycophenolate mofetil may be associated with gastrointestinal, hepatic, haematological, infectious, or reproductive adverse effects [78,79,80,81]. Prolonged immunosuppression may additionally generate concern regarding malignancy risk, although the magnitude of risk varies substantially according to the agent, cumulative exposure, patient characteristics, and concomitant risk factors. Current AAD and European-derived guidelines therefore recommend individualized selection and appropriate clinical and laboratory monitoring rather than treating conventional immunosuppressive agents as interchangeable therapies [78,79,80,81,82,83].

Targeted treatments have substantially changed the long-term management of moderate-to-severe AD, but they are not entirely free from treatment burden.

Biologic therapies targeting IL-4/IL-13 signalling have substantially improved the control of disease signs and symptoms [58]. Their potential treatment-related burden includes repeated injections and agent-specific adverse events, particularly ocular surface disease. Long-term real-world studies of dupilumab have demonstrated sustained effectiveness and a favourable safety profile for up to five years [84]. Additional real-world evidence has described the main reasons for treatment discontinuation during long-term dupilumab therapy [85].

Oral JAK inhibitors can provide rapid improvement in the signs and symptoms of moderate-to-severe AD [59]. However, their use requires consideration of infections (particularly herpes zoster), laboratory abnormalities, acne, and potential cardiovascular, thromboembolic, and malignancy risks in susceptible patients. These risks should be interpreted according to patient age, comorbidities, dose, concomitant treatments, and absolute baseline risk. An integrated analysis of phase 3 studies found no new major safety signals with extended upadacitinib exposure [82]. For abrocitinib, adverse events of special interest were more frequent among patients aged 65 years or older, supporting age- and risk-adapted patient selection and monitoring [83].

Importantly, recognition of treatment-related impairment should not lead to therapeutic inertia. Inadequately controlled AD carries substantial and potentially cumulative consequences. A CLCI-oriented approach therefore requires balancing the long-term risks of uncontrolled inflammation against the adverse effects, practical demands, monitoring requirements, and accessibility of each therapeutic option. Shared decision making, proactive management of adverse effects, adherence support, and regular reassessment of treatment goals may reduce both disease-related and treatment-related contributions to CLCI [84,85,86,87].

The present review should be considered complementary to, but distinct from, the recent review by Augustin et al. [17]. Augustin et al. provided a broad lifespan-oriented overview of CLCI in AD, focusing primarily on its recognition, measurement, prevention, and potential implementation in clinical practice. By contrast, the specific contribution of the present review is to critically examine the extent to which the CLCI framework is supported by empirical evidence. In particular, we distinguish multidimensional disease burden assessed at a single time point from evidence of persistence, recurrence, interaction, temporal accumulation, or lasting changes in a patient’s life trajectory. We also integrate quantitative evidence across psychiatric, cognitive, social, educational, occupational, economic, comorbidity, caregiver, flare-related, and treatment-related domains. Particular attention is given to recurrent flares as a potential mechanism of cumulative impairment, to the interdependent burden experienced by paediatric patients and their caregivers, and to the dual role of treatment as both a potential means of preventing CLCI and a possible contributor to cumulative burden. Therefore, the principal knowledge gap addressed by this review is not whether AD causes substantial multidimensional burden, which is already well established, but whether and how this burden accumulates over time, produces lasting consequences, and can be prevented or reversed. The available evidence supports the clinical plausibility of these processes but remains predominantly cross-sectional and rarely includes longitudinal CLCI-specific assessments.

Several limitations should be acknowledged. First, this was a narrative rather than systematic review; consequently, article selection and interpretation may be affected by selection bias, and no formal risk-of-bias assessment was performed. Second, the evidence base is heterogeneous in terms of population age, disease severity, diagnostic definitions, clinical outcomes, and follow-up duration. Third, most available studies are cross-sectional or assess isolated domains of burden rather than CLCI as an integrated longitudinal construct. Associations with psychiatric, cognitive, systemic, or socioeconomic outcomes therefore cannot establish causality. Fourth, studies using the DermCLCI instruments in AD remain scarce, and the relative contribution, temporal sequence, interaction, and reversibility of individual domains are incompletely understood. Finally, the safety profile and accessibility of systemic treatments continue to evolve, meaning that treatment-related contributions to CLCI may differ according to healthcare system, patient characteristics, and duration of exposure.

Future studies should prospectively evaluate CLCI from childhood through older adulthood, compare trajectories between paediatric- and adult-onset disease, and determine whether cumulative impairment can be prevented or reversed. Longitudinal studies should combine validated CLCI instruments with clinical severity, disease control, flare frequency, mental health, cognition, education, employment, relational outcomes, caregiver burden, treatment exposure, and healthcare costs. It will also be important to establish whether early sustained disease control modifies long-term life outcomes and to identify which patients derive the greatest overall benefit after accounting for both treatment effectiveness and treatment-related burden.

## 5. Conclusions

AD should be regarded as a life-course disease in which biological, psychological, social, contextual, and treatment-related factors interact over time to shape long-term outcomes. The available literature supports the clinical plausibility of cumulative impairment, but its incidence, progression, and reversibility have not yet been adequately established. A CLCI-oriented approach may facilitate the earlier identification of vulnerable patients and support proactive, individualized, and multidisciplinary care aimed at reducing avoidable long-term burden. However, robust longitudinal evidence and further validation of CLCI-specific instruments are required before CLCI can be routinely quantified or adopted as a validated treatment target in AD.

## Data Availability

No new data were created or analyzed in this study. Data sharing is not applicable to this article.

## References

[1] Meledathu S., Naidu M.P., Brunner P.M. (2025). Update on atopic dermatitis. J. Allergy Clin. Immunol..

[2] Savva M., Papadopoulos N.G., Gregoriou S., Katsarou S., Papapostolou N., Makris M., Xepapadaki P. (2024). Recent advancements in the atopic dermatitis mechanism. Front. Biosci..

[3] Napolitano M., Megna M., Patruno C., Gisondi P., Ayala F., Balato N. (2016). Adult atopic dermatitis: A review. G. Ital. Dermatol. Venereol..

[4] Maurelli M., Chiricozzi A., Peris K., Gisondi P., Girolomoni G. (2023). Atopic dermatitis in the elderly population. Acta Derm. Venereol..

[5] Facheris P., Correa Da Rosa J., Pagan A.D., Angelov M., Del Duca E., Rabinowitz G., Gómez-Arias P.J., Rothenberg-Lausell C.D., Estrada Y.D., Bose S. (2023). Age of onset defines two distinct profiles of atopic dermatitis in adults. Allergy.

[6] Basra M.K.A., Salek M.S., Finlay A.Y. (2012). The Dermatology Life Quality Index (DLQI): A fundamental measure of patient-reported outcomes in dermatology. Dermatol. Clin..

[7] Silverberg J.I. (2017). Public Health Burden and Epidemiology of Atopic Dermatitis. Dermatol. Clin..

[8] Drucker A.M., Wang A.R., Li W.Q., Sevetson E., Block J.K., Qureshi A.A. (2017). The Burden of Atopic Dermatitis: Summary of a Report for the National Eczema Association. J. Innov. Dermatol..

[9] Bawany F., Northcott C.A., Beck L.A., Pigeon W.R. (2021). Sleep Disturbances and Atopic Dermatitis: Relationships, Methods for Assessment, and Therapies. J. Allergy Clin. Immunol. Pract..

[10] Whiteley J., Emir B., Seitzman R., Makinson G. (2016). The burden of atopic dermatitis in US adults: Results from the 2013 National Health and Wellness Survey. Curr. Med. Res. Opin..

[11] Eckert L., Gupta S., Amand C., Gadkari A., Mahajan P., Gelfand J.M. (2017). Impact of atopic dermatitis on health-related quality of life and productivity in adults in the United States: An analysis using the National Health and Wellness Survey. J. Am. Acad. Dermatol..

[12] Gwillim E.C., Nattkemper L., Yosipovitch G. (2021). Impact of Itch on Sleep Disturbance in Patients with Prurigo Nodularis. Acta Derm. Venereol..

[13] Eckert L., Gupta S., Amand C., Gadkari A., Mahajan P., Gelfand J.M. (2018). The burden of atopic dermatitis in US adults: Health care resource utilization data from the 2013 National Health and Wellness Survey. J. Am. Acad. Dermatol..

[14] Yang E.J., Beck K.M., Sekhon S., Bhutani T., Koo J. (2019). The impact of pediatric atopic dermatitis on families: A review. Pediatr. Dermatol..

[15] Vakharia P.P., Cella D., Silverberg J.I. (2018). Patient-reported outcomes and quality of life measures in atopic dermatitis. Clin. Dermatol..

[16] Braren-von Stülpnagel C.C., Augustin M., Westphal L., Sommer R. (2021). Mapping risk factors for cumulative life course impairment in patients with chronic inflammatory skin diseases: A systematic review. J. Eur. Acad. Dermatol. Venereol..

[17] Augustin M., Amerio P., Bewley A., Gieler U., Gutermuth J., Kimball A.B., Simpson E., Warren R.B. (2026). Atopic Dermatitis Across the Lifespan: Understanding, Measuring and Minimizing Cumulative Life Course Impairment. Acta Derm. Venereol..

[18] Wolf J., Anstey A.V., Chin M.F. (2013). Evaluation of patient experience of atopic dermatitis with a view of assessing whether life course impairment is cumulative. J. Am. Acad. Dermatol..

[19] Braren-von Stülpnagel C.C., Augustin M., Westphal L., Sommer R. (2023). Development of Measurement Tools to Assess Cumulative Life Course Impairment in Patients with Chronic Skin Diseases. J. Eur. Acad. Dermatol. Venereol..

[20] Ho M.J., Sultana R., Choo K., Lee H.Y. (2025). Cumulative Life Course Impairment in Moderate-to-Severe Atopic Dermatitis: A Cross-Sectional Study in Sin-gapore. Dermatology.

[21] Carroll C.L., Balkrishnan R., Feldman S.R., Fleischer A.B., Manuel J.C. (2005). The burden of atopic dermatitis: Impact on the patient, family, and society. Pediatr. Dermatol..

[22] Ravnborg N., Ambikaibalan D., Agnihotri G., Price S., Rastogi S., Patel K.R., Singam V., Andersen Y., Halling A.-S., Silverberg J.I. (2021). Prevalence of asthma in patients with atopic dermatitis: A systematic review and meta-analysis. J. Am. Acad. Dermatol..

[23] Knudgaard M.H., Andreasen T.H., Ravnborg N., Bieber T., Silverberg J.I., Egeberg A., Halling A.-S., Thyssen J.P. (2021). Rhinitis prevalence and association with atopic dermatitis: A systematic review and meta-analysis. Ann. Allergy Asthma Immunol..

[24] Flohr C., Perkin M., Logan C., Marrs T., Radulovic S., Campbell L.E., MacCallum S.F., McLean W.H.I., Lack G. (2014). Atopic dermatitis and disease severity are the main risk factors for food sensitization in exclusively breastfed infants. J. Investig. Dermatol..

[25] Halvorsen J.A., Lien L., Dalgard F., Bjertness E., Stern R.S. (2014). Suicidal ideation, mental health problems and social functioning in adolescents with eczema. J. Investig. Dermatol..

[26] Dieris-Hirche J., Gieler U., Petrak F., Milch W., te Wildt B., Dieris B., Herpertz S. (2017). Suicidal ideation in adult patients with atopic dermatitis. Acta Derm. Venereol..

[27] Andersen L., Nyeland M.E., Nyberg F. (2020). Increasing severity of atopic dermatitis is associated with a negative impact on work productivity and activity impairment. Br. J. Dermatol..

[28] Stratigos A.J., Chasapi V., Katoulis A., Vakirlis E., Psarros F., Georgiou S., Vourdas D., Makris M., Lazaridou E., Gregoriou S. (2024). Unveiling the Impact of Moderate and Severe Atopic Dermatitis: Insights on Burden, Clinical Characteristics, and Healthcare Resource Utilization in Adult Greek Patients from the APOLO Cross-Sectional Study. J. Clin. Med..

[29] Napolitano M., Fabbrocini G., Kastl S., Battista T., Di Guida A., Martora F., Picone V., Ventura V., Patruno C. (2022). Effect of dupilumab on sexual desire in adult patients with moderate-to-severe atopic dermatitis. Medicina.

[30] Misery L., Seneschal J., Corgibet F., Halioua B., Sampogna F., Merhand S., Skayem C., Ben Hayoun Y., Taieb C., Staumont-Sallé D. (2025). Sexual impairment in patients with atopic dermatitis. Clin. Exp. Dermatol..

[31] Rodriguez-Pozo J.-A., Montero-Vilchez T., Diaz Calvillo P., Sanabria de la Torre R., Ureña-Paniego C., Ramirez-Muñoz A., Arias-Santiago S. (2024). The Impact of Atopic Dermatitis on Sexual Function and Reproductive Desires in Women. Acta Derm. Venereol..

[32] Silverberg J.I., Dodiuk-Gad R., Takaoka R., Tan J., Gu C., Luger T., Halioua B., Aslanian F., Prakoeswa C.R.S., Demessant Flavigny A.-L. (2025). Atopic dermatitis starting in childhood has a stronger impact on adults: Results from the ’Scars of Life’ international project in 27 countries. Br. J. Dermatol..

[33] Ständer S., Weisshaar T., Mettang T., Szepietowski J.C., Carstens E., Ikoma A., Bergasa N.V., Gieler U., Misery L., Wallengren J. (2007). Clinical classification of itch: A position paper of the International Forum for the Study of Itch. Acta Derm. Venereol..

[34] Mollanazar N.K., Smith P.K., Yosipovitch G. (2016). Mediators of chronic pruritus in atopic dermatitis. Allergy.

[35] Pereira M.P., Kremer A.E., Mettang T., Ständer S. (2016). Chronic Pruritus in the Absence of Skin Disease: Pathophysiology, Diagnosis and Treatment. Am. J. Clin. Dermatol..

[36] Yosipovitch G., Misery L., Proksch E., Metz M., Ständer S., Schmelz M. (2019). Skin Barrier Damage and Itch: Review of Mechanisms, Topical Management and Future Directions. Acta Derm. Venereol..

[37] Silverberg J.I., Gelfand J.M., Margolis D.J., Boguniewicz M., Fonacier L., Grayson M.H., Simpson E.L., Ong P.Y., Chiesa Fuxench Z.C. (2018). Patient burden and quality of life in atopic dermatitis in US adults. Ann. Allergy Asthma Immunol..

[38] Yu S.H., Attarian H., Zee P., Silverberg J.I. (2016). Sleep disturbance in adults with atopic dermatitis: A systematic review. J. Am. Acad. Dermatol..

[39] Silverberg J.I. (2015). Association between atopic dermatitis and sleep disorders in US adults. J. Investig. Dermatol..

[40] Chang Y.S., Chiang B.L. (2018). Sleep disorders and atopic dermatitis: A 2-way relationship. Allergy Asthma Immunol. Res..

[41] Hurel Barroso M., Oliveira Rodrigues Valle S., Duarte Dortas Junior S., Raggio Luiz R., Lupi O. (2024). Sleep and quality of life in adults with atopic dermatitis: Cross sectional study. Eur. Ann. Allergy Clin. Immunol..

[42] Itani O., Jike M., Watanabe N., Kaneita Y. (2017). Short sleep duration and health outcomes: A systematic review, meta-analysis, and meta-regression. Sleep Med..

[43] Ghanem F.K., Attarian H., Al-Khalil Z., Kabrita C.S. (2026). Sleep loss as a cardiometabolic risk factor: A narrative review of clinical and public health implications. J. Clin. Sleep Med..

[44] Mullington J.M., Simpson N.S., Meier-Ewert H.K., Haack M. (2010). Sleep loss and inflammation. Best Prac. Res. Clin. Endocrinol. Metab..

[45] Baglioni C., Nanovska S., Regen W., Spiegelhalder K., Feige B., Nissen C., Reynolds C.F., Riemann D. (2016). Sleep and mental disorders: A meta-analysis of polysomnographic research. Psychol. Bull..

[46] Fang H., Tu S., Sheng J., Shao A. (2019). Depression in sleep disturbance: A review on a bidirectional relationship, mechanisms and treatment. J. Cell. Mol. Med..

[47] Goodman M.L., Lee M., Springer A., Schick V., Vaughan E., Markham C., Gitari S., Mukiri F. (2024). Sleep disturbance as a precursor to anxiety, depression, and PTSD among rural Kenyans: A cross-lagged panel analysis from a rural Kenyan interventional cohort. J. Sleep Res..

[48] Rønnstad A.T.M., Halling-Overgaard A.-S., Hamann C.R., Skov L., Egeberg A., Thyssen J.P. (2018). Association of atopic dermatitis with depression, anxiety, and suicidal ideation: A systematic review and meta-analysis. J. Am. Acad. Dermatol..

[49] Kage P., Poblotzki L., Zeynalova S., Zarnowski J., Simon J.C., Treudler R. (2022). Depression, Anxiety, and Suicidal Ideation in Patients with Atopic Eczema in a Prospective Study in Leipzig, Germany. Int. Arch. Allergy Immunol..

[50] Treudler R., Zeynalova S., Riedel-Heller S.G., Zuelke A.E., Roehr S., Hinz A., Glaesmer H., Kage P., Loeffler M., Simon J.C. (2020). Depression, anxiety and quality of life in subjects with atopic eczema in a population-based cross-sectional study in Germany. J. Eur. Acad. Dermatol. Venereol..

[51] Bialas A., Rabe M., Artz N., Boldt A., Simon J.C., Treudler R., Klein B. (2025). Atopic Multimorbidity in Adults with a Focus on Sensitization Patterns and T Cell Activation. Clin. Transl. Allergy.

[52] Zhou Q., Yang D., Xiong C., Li X. (2023). Atopic dermatitis and cognitive dysfunction in middle-aged and older adults: A systematic review and me-ta-analysis. PLoS ONE.

[53] Halioua B., Skayem C., Merhand S., Hayoun Y.B., Taieb C. (2024). Long-term consequences on stigmatization and disease burden during adulthood among patients with childhood or adolescence-onset atopic dermatitis. Br. J. Dermatol..

[54] Adamson A.S., Feldman S.R., Strowd L.C., Lovell K.K. (2024). The economic impact of atopic dermatitis. Management of Atopic Dermatitis.

[55] Sach T.H., McManus E., Levell N.J. (2019). Understanding economic evidence for the prevention and treatment of atopic eczema. Br. J. Dermatol..

[56] Beikert F.C., Langenbruch A.K., Radtke M.A., Kornek T., Purwins S., Augustin M. (2014). Willingness to pay and quality of life in patients with atopic dermatitis. Arch. Dermatol. Res..

[57] Chovatiya R., Begolka W.S., Thibau I.J., Silverberg J.I. (2022). Impact and associations of atopic dermatitis out-of-pocket health care expenses in the United States. Dermatitis.

[58] Simpson E.L., Bieber T., Guttman-Yassky E., Beck L.A., Blauvelt A., Cork M.J., Silverberg J.I., Deleuran M., Kataoka Y., Lacour J.-P. (2016). Two phase 3 trials of dupilumab versus placebo in atopic dermatitis. N. Engl. J. Med..

[59] Reich K., Teixeira H.D., de Bruin-Weller M., Bieber T., Soong W., Kabashima K., Werfel T., Zeng J., Huang X., Hu X. (2021). Safety and efficacy of upadacitinib in combination with topical corticosteroids in adolescents and adults with moderate-to-severe atopic dermatitis (AD Up): Results from a randomised, double-blind, placebo-controlled, phase 3 trial. Lancet.

[60] Thyssen J.P., Halling A.S., Schmid-Grendelmeier P., Guttman-Yassky E., Silverberg J.I. (2023). Comorbidities of atopic dermatitis—What does the evidence say?. J. Allergy Clin. Immunol..

[61] Christensen M.O., Barakji Y.A., Loft N., Khatib C.M., Egeberg A., Thomsen S.F., Silverberg J.I., Flohr C., Maul J.T., Schmid-Grendelmeier P. (2023). Prevalence of and association between atopic dermatitis and food sensitivity, food allergy and challenge-proven food allergy: A systematic review and meta-analysis. J. Eur. Acad. Dermatol. Venereol..

[62] Jensen M.B., Kursawe Larsen C., Hamann C.R., Johansen J.D., Quaade A.S. (2026). Association Between Atopic Dermatitis and Contact Sensitization: An Updated Systematic Review and Meta-Analysis. Contact Dermat..

[63] Silverberg J.I. (2019). Comorbidities and the impact of atopic dermatitis. Ann. Allergy Asthma Immunol..

[64] Kong H.H., Oh J., Deming C., Conlan S., Grice E.A., Conlan S., Grice E.A., Beatson M.A., Nomicos E., Polley E.C. (2012). Temporal shifts in the skin microbiome associated with disease flares and treatment in children with atopic dermatitis. Genome Res..

[65] Totté J.E., van der Feltz W.T., Hennekam M., van Belkum A., van Zuuren E.J., Pasmans S.G. (2016). Prevalence and odds of Staphylococcus aureus carriage in atopic dermatitis: A systematic review and meta-analysis. Br. J. Dermatol..

[66] Weidinger S., Novak N. (2016). Atopic dermatitis. Lancet.

[67] Legat F.J. (2021). Itch in Atopic Dermatitis—What Is New?. Front. Med..

[68] Kimball A.B., Gieler U., Linder D., Sampogna F., Warren R.B., Augustin M. (2010). Psoriasis: Is the impairment to a patient’s life cumulative?. J. Eur. Acad. Dermatol. Venereol..

[69] Gulliver W.P. (2011). Cumulative life course impairment in psoriasis: Patient perception of disease-related impairment throughout the life course. Br. J. Dermatol..

[70] Mattei P.L., Corey K.C., Kimball A.B. (2013). Cumulative life course impairment: Evidence for psoriasis. Curr. Probl. Dermatol..

[71] Romiti R., Magalhães R.F., Duarte G.V. (2024). Cumulative life course impairment in patients with dermatological diseases, with a focus on psoriasis. Bras. Dermatol..

[72] Sampogna F., Tabolli S., Abeni D. (2013). Impact of different skin conditions on quality of life. G. Ital. Dermatol. Venereol..

[73] Yap J.C.H., Yew Y.W. (2024). Impact of Atopic Dermatitis^®^ on Quality of Life of Caregivers: A Systematic Review and Meta-Analysis. Dermatitis.

[74] Harvey J., Lax S.J., Lowe A., Santer M., Lawton S., Langan S.M., Roberts A., Stuart B., Williams H.C., Thomas K.S. (2023). The long-term safety of topical corticosteroids in atopic dermatitis: A systematic review. Ski. Health Dis..

[75] Argenziano G., Cusano F., Corazza M., Amato S., Amerio P., Naldi L., Patruno C., Pigatto P.D., Quaglino P., Gisondi P. (2024). Italian S3-Guideline on the treatment of Atopic Eczema—Part 1: Systemic therapy, adapted from EuroGuiDerm by SIDEMAST, ADOI and SIDAPA. Ital. J. Dermatol. Venerol..

[76] Davis D.M.R., Drucker A.M., Alikhan A., Bercovitch L., Cohen D.E., Darr J.M., Eichenfield L.F., Frazer-Green L., Paller A.S., Schwarzenberger K. (2024). Guidelines of care for the management of atopic dermatitis in adults with phototherapy and systemic therapies. J. Am. Acad. Dermatol..

[77] Yu S.H., Drucker A.M., Lebwohl M., Silverberg J.I. (2018). A systematic review of the safety and efficacy of systemic corticosteroids in atopic dermatitis. J. Am. Acad. Dermatol..

[78] Napolitano M., Fabbrocini G., Potestio L., Fontanella G., Picone V., Bennardo L., Scalvenzi M., Patruno C. (2022). A 24-weeks real-world experience of dupilumab in adolescents with moderate-to-severe atopic dermatitis. Dermatol. Ther..

[79] Potestio L., Napolitano M., Bennardo L., Fabbrocini G., Patruno C. (2022). Atopic dermatitis exacerbation after Covid-19 vaccination in Dupilumab-treated patients. J. Eur. Acad. Dermatol. Venereol..

[80] Napolitano M., Fabbrocini G., Genco L., Martora F., Potestio L., Patruno C. (2022). Rapid improvement in pruritus in atopic dermatitis patients treated with upadacitinib: A real-life experience. J. Eur. Acad. Dermatol. Venereol..

[81] Napolitano M., Maffei M., Patruno C., Leone C.A., Di Guida A., Potestio L., Scalvenzi M., Fabbrocini G. (2021). Dupilumab effectiveness for the treatment of patients with concomitant atopic dermatitis and chronic rhinosinusitis with nasal polyposis. Dermatol. Ther..

[82] Guttman-Yassky E., Thyssen J.P., Silverberg J.I., Papp K.A., Paller A.S., Weidinger S., Hong H.C.-h., Hendrickson B., Dilley D., Tenorio A.R. (2023). Safety of upadacitinib in moder-ate-to-severe atopic dermatitis: An integrated analysis of phase 3 studies. J. Allergy Clin. Immunol..

[83] Cork M.J., Deleuran M., Geng B., Silverberg J.I., Simpson E.L., Stein Gold L.F., Irvine A.D., Romero W., Valdez H., Fan H. (2025). Long-Term Safety of Abrocitinib in Moder-ate-to-Severe Atopic Dermatitis: Integrated Analysis by Age. J. Allergy Clin. Immunol. Pract..

[84] Zhang J., Boesjes C.M., Loman L., Kamphuis E., Romeijn M.L.E., Spekhorst L.S., Haeck I., van der Gang L.F., Dekkers C.C., van der Rijst L.P. (2024). Dupilumab provides sustained effectiveness on patient-reported outcomes and favorable safety in patients with moderate-to-severe atopic dermatitis: Up to 5-year results from the daily practice BioDay registry. J. Am. Acad. Dermatol..

[85] Boesjes C.M., Kamphuis E., de Graaf M., Spekhorst L.S., Haeck I., van der Gang L.F., Loman L., Zuithoff N.P.A., Dekkers C., van der Rijst L.P. (2024). Long-Term Effectiveness and Reasons for Discontinuation of Dupilumab in Patients with Atopic Dermatitis. J. Am. Acad. Dermatol..

[86] Boostani M., Bánvölgyi A., Goldust M., Cantisani C., Pietkiewicz P., Lőrincz K., Holló P., Wikonkál N.M., Paragh G., Kiss N. (2025). Diagnostic Performance of GPT-4o and Gemini Flash 2.0 in Acne and Rosacea. Int. J. Dermatol..

[87] Boostani M., Bánvölgyi A., Zouboulis C.C., Goldfarb N., Suppa M., Goldust M., Lőrincz K., Kiss T., Nádudvari N., Holló P. (2025). Large language models in evaluating hidradenitis suppurativa from clinical images. J. Eur. Acad. Dermatol. Venereol..

